# Shall We Focus on the Eosinophil to Guide Treatment with Systemic Corticosteroids during Acute Exacerbations of COPD?: PRO

**DOI:** 10.3390/medsci6030074

**Published:** 2018-09-11

**Authors:** James Camp, Jennifer L. Cane, Mona Bafadhel

**Affiliations:** 1 Respiratory Medicine Unit, Nuffield Department of Clinical Medicine, University of Oxford, Oxford OX3 7FZ, UK; jamescamp438@gmail.com (J.C.); jennifer.cane@ndm.ox.ac.uk (J.L.C.); 2 NIHR Oxford Biomedical Research Centre, University of Oxford, Oxford OX3 7FZ, UK

**Keywords:** COPD, Eosinophils, Inflammation

## Abstract

In an era of precision medicine, it seems regressive that we do not use stratified approaches to direct treatment of oral corticosteroids during an exacerbation of chronic obstructive pulmonary disease (COPD). This is despite evidence suggesting that 40% of COPD patients have eosinophilic inflammation and this is an indicator of corticosteroid response. Treatments with oral corticosteroids are not always effective and not without harm, with significant and increased risk of hyperglycemia, sepsis, and fractures. Eosinophils are innate immune cells with an incompletely understood role in the pathology of airway disease. They are detected at increased levels in some patients and can be measured using non-invasive methods during states of exacerbation and stable periods. Despite the eosinophil having an unknown mechanism in COPD, it has been shown to be a marker of length of stay in severe hospitalized exacerbations, a predictor of risk of future exacerbation and exacerbation type. Although limited, promising data has come from one prospective clinical trial investigating the eosinophil as a biomarker to direct systemic corticosteroid treatment. This identified that there were statistically significant and clinically worsened symptoms in patients with low eosinophil levels who were prescribed prednisolone, demonstrating the potential utility of the eosinophil. In an era of precision medicine our patients’ needs are best served by accurate diagnosis, correct identification of maximal treatment response and the abolition of harm. The peripheral blood eosinophil count could be used towards reaching these aims.

## 1. Eosinophil Cell Biology

Eosinophils are inflammatory leukocytes comprising of bi-lobed nuclei and large acidophilic cytoplasm granules. The cationic protein granules bind to acid stains and in particular eosin which allowed them to be identified by Paul Ehrlich in 1879 [1]. Four proteins are found to make up the granules including major basic protein (MBP) located in the core and eosinophil basic proteins forming the matrix, consisting of eosinophil cationic protein (ECP), eosinophil peroxidase (EPO), and eosinophil-derived neurotoxin (EDN). These granules are toxic to various tissues and are capable of inducing damage and dysfunction upon their secretion [2].

Produced in healthy bone marrow derived from CD34+ myeloid progenitors, the number of eosinophils generated is typically low with circulating eosinophils range between 1–4% of the total white blood cell count. Once mature, eosinophils enter the systemic circulation where they can reside for 8–12 h. Unless stimulated, the cells then migrate to tissues [3]. Differentiation for this lineage is promoted by interleukin (IL)-3, granulocyte/macrophage-colony-stimulating factor (GM-CSF) and IL-5 cytokines from a hematopoietic stem cell into a mature eosinophil [4]. These same cytokines also act as priming agents, transforming the eosinophil from a quiescent cell into a hyper-responsive state. This includes an increased response to chemotaxis, degranulation, and cytokine production [5]. The mechanism of entry of the eosinophil from the blood stream into tissue involves crossing a microvascular wall. Eotaxin (CCL11) and IL-5 are the two main ligands that promote eosinophil migration into the tissue [6]. The Eotaxins, in addition to RANTES, produced from epithelial, mesenchymal, and endothelial cells are involved in migration and priming of eosinophils once in the airway [7,8]. Expression of C-C chemokine receptor type 3 (CCR3) and IL-5 receptor subunit alpha (IL5Rα) on the cell surface is integral to eosinophil recruitment to tissues. The role of the eosinophil in both innate and adaptive immunity is poorly defined, and their response varies depending on the environment and/or stimulus. Resident eosinophils are predominately found in the gastrointestinal tract, although other resident populations are also found in healthy individuals in physiological conditions in the thymus, spleen, lymph nodes, mammary glands and the uterus indicating other potential roles in homeostasis. Their accumulation at these sites has given rise to the eosinophil role extending to local immunity and/or remodeling and repair in health and disease (the so called “LIAR” hypothesis) [9].

**Figure 1:**
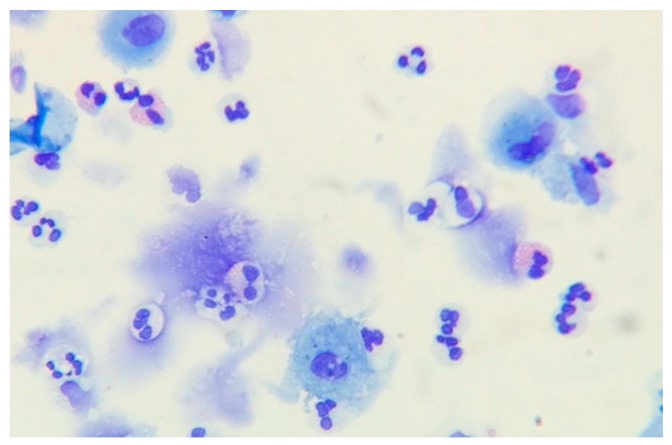
Sputum slide showing eosinophils.

Eosinophils play an important role in immune-regulation by priming B cells and maintaining type-2 immunity [10]. In the airway, the eosinophil can act as an antigen-presenting cell while several stored and secreted mediators highlight their role as both an immunomodulatory and effector cell in the airway (Figure 1) [11]. Eosinophils are capable of disrupting the pulmonary epithelial barrier and causing alveolar epithelium cell lysis upon granular secretion, further exacerbating the inflammatory response [10].

## 2. Eosinophils in Chronic Obstructive Pulmonary Disease

In asthma, another common airway disease, the utility of the eosinophil to identify a corticosteroid response has been established [12], leading the way to the successful development of monoclonal antibodies to target severe eosinophilic asthma [13,14,15,16,17]. The lack of an underlying mechanism for the role of eosinophils in asthma has not diminished their use in clinical practice to identify the patient that requires treatment with anti-eosinophil depleting treatments, such as inhaled or oral corticosteroids, or monoclonal antibodies.

The involvement of the eosinophil in the pathogenesis of chronic obstructive pulmonary disease (COPD) has not been fully elucidated [18] and their role remains controversial. Up to 40% of patients with COPD have eosinophilic airway inflammation and both invasive and non-invasive methods have been used when measuring for this phenotype [19]. Historically, measurements of sputum eosinophils have been undertaken to categorize the degree of eosinophilic inflammation found in patients with COPD [20]. Although sputum induction is both a safe and repeatable procedure [20], it requires time, technical processing and expertise in slide preparation and counting [21]. Hence, sputum measurements in COPD (nor asthma) have not been adopted in routine clinical practice. The peripheral blood eosinophil count has emerged as an ideal surrogate for sputum eosinophilic inflammation [22]. Near-patient testing, validated against standard venipuncture laboratory analysis [23] allows for the rapid measurement of the peripheral blood eosinophil count in real-time and in the clinic room or surgery. In stable COPD, sputum eosinophils have been shown to identify both inhaled [24] and oral corticosteroid [25] response with respect to lung function improvements, quality of life and exercise capacity. Furthermore, in a direct replica of the seminal asthma study by Green et al. [12], reduction of sputum eosinophils in stable COPD has been shown to reduce exacerbations in the order of 65% [26].

Since the emergence of the peripheral blood eosinophil as a useful biomarker in COPD, several post-hoc analysis have explored its utility in directing inhaled corticosteroids to impact on exacerbation burden [27,28,29,30]. These studies have unequivocally found that in patients with COPD and a history of exacerbations, the peripheral blood eosinophil identifies patients with an increased risk of exacerbations and the best response to inhaled corticosteroids [27,28,29] or a worsened response to withdrawal of inhaled corticosteroids [31,32]. It is thus conceivable and arguably plausible that the measurement of eosinophilic inflammation is likely to be crucial in determining the phenotype of the disease and direction of therapy, making it critically useful as a biomarker to aid understanding in COPD pathogenesis and treatment response [18].

## 3. Eosinophils in Exacerbations of COPD

Exacerbations of COPD are heterogeneous and attempts to delineate the biological heterogeneity using plasma markers and symptoms have been made [33]. Unsupervised cluster analysis of sputum mediators has highlighted that there are independent biological clusters, which relate to unique inflammatory pathways and to underlying pathogenic etiology [22]. The biology of the exacerbation varies with the presence of bacteria, virus, eosinophils and in some a low inflammatory state (likely because of cardiac dysfunction or co-morbidity) [22]. The utility of the peripheral blood eosinophil as a suitable, sensitive and specific biomarker in COPD and in particular COPD exacerbations, identifying a type-2 eosinophilic phenotype was first reported in 2011 [22] and has been further validated by other groups [34]. Exacerbations of COPD that are associated with a type-2 inflammatory response have been shown to have increased concentrations of IL-5 and CCL11 and increased concentrations of sputum and blood eosinophils [22]. Furthermore, the clinical characteristics at the onset of an exacerbation cannot distinguish either type-1 or type-2 immune response [22,35,36]. Measurements of biological expression during an exacerbation of COPD could be used to stratify treatment.

## 4. Systemic Corticosteroids at the Onset of an Exacerbation: A Poorly Effective Treatment?

At present, there is no ideal method to direct treatment during an exacerbation of COPD. In the presence of dyspnea, exacerbations are usually always treated with systemic corticosteroids [37]. This is despite a small number of patients studied and heterogeneous evidence [37,38,39]. These treatments are routinely given in attempt to improve patient outcomes such as symptom recovery and prevent a treatment failure (defined as re-treatment, hospitalization or death), but have no effect on length of intensive treatment unit (ITU) stay or longer term lung function and are not without harm [40,41,42,43]. A Cochrane review for systemic corticosteroids in the management of an exacerbation of COPD demonstrates no effect on mortality and a small reduction in treatment failures, with a number-need-to-treat of 10, but a number-needed-to-harm of 6 [42]. Approximately 1 in 13 patients with an exacerbation of COPD treated with systemic corticosteroids will develop significant hyperglycemia [42]. A recent retrospective cohort case-control study in the emergency department demonstrates that even one short course of systemic corticosteroids are associated with an increase rate of sepsis (incidence rate ratio 5.3, 95% confidence interval (CI) 3.8–7.4), venous thromboembolism (incidence rate ratio 3.3, 95% CI 2.8–4.0) and fractures (incidence rate ratio 1.9, 95% CI 1.7–2.1) [40]. Corticosteroids however are an effective yet non-specific anti-inflammatory [44]. Approximately 30% of exacerbations of COPD are associated with eosinophilic airway inflammation [22]. Exacerbations of COPD with sputum eosinophilia have been shown to have the best forced expiratory volume (FEV1) response to systemic corticosteroid therapy [45]. Despite their unknown mechanism in airways disease such as asthma and COPD, the eosinophil does inform of the likelihood of response to corticosteroids.

In a post-hoc analysis from severe hospitalized exacerbations, length of hospital stay is significantly shorter in eosinophilic exacerbations treated with systemic corticosteroids [46] while retrospective analysis in the intensive care unit, demonstrates that eosinophilic exacerbations are associated with a lower mortality in COPD patients which are invasively ventilated [47]. Finally, the corroboration that eosinophils are an important biomarker in COPD exacerbations is demonstrated with the DECAF index, with eosinophils (and low levels, below 50 cells/mm^3^) being an independent predictor of mortality in severe hospitalized exacerbations of COPD [48].

## 5. Eosinophils at the Onset of an Exacerbation to Direct Prednisolone Treatment: Time to Move Towards Precision

Clinical trials using biomarkers such as Procalcitonin [49] or C-reactive protein [50] have been successful in determining antibiotic prescription in exacerbations of COPD and go to some lengths to drive antibiotic stewardship [51,52]. In the only prospective study so far, the eosinophil has been successfully used to direct systemic corticosteroids at the time of a moderate exacerbation of COPD [53]. This single-center proof of concept study reached its primary outcome of non-inferiority in treatment failure rates in biomarker-directed treatment of systemic corticosteroids versus standard therapy with the additional signal of increased harm in patients who had low eosinophil levels (“biomarker low” peripheral blood eosinophil counts below 2%) prescribed prednisolone. Furthermore, the “biomarker low” patient population which received prednisolone therapy reported both statistically significant and clinically worsened symptoms and a slower rate of recovery than in “biomarker low” patient population receiving placebo. These findings were almost replicated in a pooled analysis of studies with available eosinophil results at the time of an exacerbation, with the worst outcomes (significantly higher treatment failure rates) in patients with eosinophilic exacerbations and treatment with placebo [54]. This has the implication that prednisolone is causing harm in some patients with COPD. At this current time there are a further two multi-center randomized trials seeking to validate this finding (NCT02857842 [55] and ISRCTN27510582), with planned read-outs by 2020.

In an era of precision medicine [56], our patients’ needs are best served by accurate diagnosis, correct identification of maximal treatment response and the abolition of harm. The peripheral blood eosinophil count could be used towards reaching these aims.
